# MethMarker: user-friendly design and optimization of gene-specific DNA methylation assays

**DOI:** 10.1186/gb-2009-10-10-r105

**Published:** 2009-10-05

**Authors:** Peter Schüffler, Thomas Mikeska, Andreas Waha, Thomas Lengauer, Christoph Bock

**Affiliations:** 1 Max-Planck-Institut für Informatik, Campus E1.4, 66123 Saarbrücken, Germany; 2 Molecular Pathology Research and Development Laboratory, Department of Pathology, Peter MacCallum Cancer Centre, A'Beckett Street, Melbourne, Victoria 8006, Australia; 3 Department of Neuropathology, University of Bonn Medical Centre, Sigmund-Freud-Straße, 53105 Bonn, Germany

## Abstract

DNA methylation is a key mechanism of epigenetic regulation that is frequently altered in diseases such as cancer. To confirm the biological or clinical relevance of such changes, gene-specific DNA methylation changes need to be validated in multiple samples. We have developed the MethMarker http://methmarker.mpi-inf.mpg.de/ software to help design robust and cost-efficient DNA methylation assays for six widely used methods. Furthermore, MethMarker implements a bioinformatic workflow for transforming disease-specific differentially methylated genomic regions into robust clinical biomarkers.

## Abstract 2

A software workflow to translate known differentially methylated regions into clinical biomarkers

## Rationale

Aberrant DNA methylation is a common event in many cancers [1,2]. Functionally, cancer-specific hypermethylation imposes condensed chromatin structure upon CpG islands that normally exhibit an open and transcriptionally competent chromatin structure [3]. This epigenetic alteration results in loss of expression at nearby genes, contributing to cancer development when tumor suppressor genes are affected [4].

For many years, research in cancer epigenetics has focused on the use of CpG island hypermethylation events of certain genes as cancer biomarkers, with the aim of improving cancer treatment through more accurate diagnosis, prognosis and therapy selection [5,6]. Early diagnosis exploits the fact that CpG island hypermethylation of cancer-related genes is frequently detectable in early-stage tumors [7], for which surgical treatment can be highly effective. Prognosis of clinical outcome uses DNA hypermethylation events to infer whether or not a tumor is likely to constitute a major threat to the patient's health, which is particularly relevant for cancers that will kill only a subset of patients if left untreated (for example, prostate cancer). Therapy optimization makes use of DNA methylation differences between patient subgroups in order to select the most effective treatment, thus contributing to personalized cancer treatment.

In spite of significant investment in genome-wide screening and subsequent validation studies, few DNA methylation biomarkers have been confirmed by clinical trials. This bottleneck in the process of translating basic research findings into the clinic is partially due to a discontinuity of methods between the discovery phase and the validation phase. The methods used most commonly in the discovery phase (such as tiling microarray and clonal bisulfite sequencing) are too time-consuming and expensive to be used in the clinical setting. Hence, candidate biomarkers have to be adapted to high sample-throughput methods such as MethyLight [8], bisulfite pyrosequencing [9-11], COBRA (combined bisulfite restriction analysis) [12] or bisulfite single nucleotide primer extension (SNuPE) [13,14]. To be effective, this adaptation step requires substantial bioinformatic optimization and validation.

**Figure 1:**
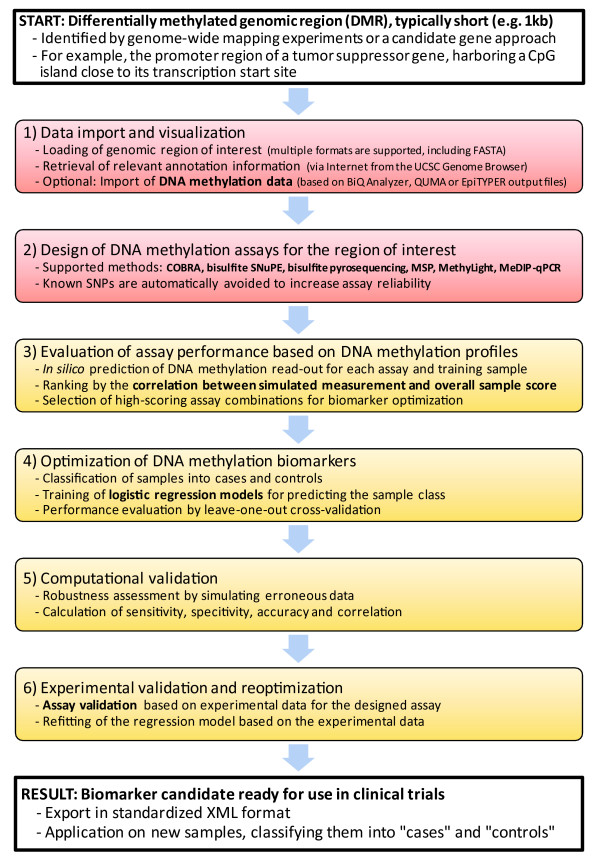
**MethMarker employs a six-step workflow to design, optimize and validate DNA methylation biomarkers for a given differentially methylated DNA region (DMR)**. In addition to its main purpose as a full-scale biomarker development tool, MethMarker can also be used simply as an assay design software, in which case steps 3 to 6 (yellow boxes) are omitted.

Based on our experience from a pilot study on the *O*^6^-methylguanine DNA methyltransferase (*MGMT*) gene [15], we have developed a systematic workflow for design, optimization and validation of DNA methylation biomarkers (reviewed in [16]). The six-step procedure outlined in Figure 1 starts from a preselected differentially methylated region (DMR), which may have been identified by genome-wide screening experiments or through a candidate gene approach. A typical example would be a CpG island that overlaps with the promoter region of a tumor suppressor gene. In the first step, this region is subjected to high-resolution analysis of DNA methylation in a small number of cases and controls (for example, by clonal bisulfite sequencing). These experimental data provide MethMarker with a representative map of methylation state within the DMR and inform all subsequent optimization steps. Second, using sets of expert rules, technically feasible DNA methylation assays are designed for each of six robust and cost-efficient experimental protocols (COBRA, bisulfite SNuPE, bisulfite pyrosequencing, MethyLight, methylation-specific polymerase chain reaction (MSP) and methylated DNA immunoprecipitation quantitative PCR (MeDIP-qPCR)). Third, the accuracy of all designed assays is computationally assessed, using the DNA methylation map derived in the first step. Fourth, the most promising candidate biomarkers are statistically optimized for maximum discrimination between cases and controls. Fifth, to reduce the risk that candidate biomarkers subsequently fail due to technical problems or lack of robustness, all high-scoring assays are validated with respect to their susceptibility to experimental noise, measurement errors and unknown single nucleotide polymorphisms. Sixth, the most promising assay is selected, experimentally tested and further optimized based on the outcome of the experimental validation. After completion of these six steps, the candidate biomarker is ready for application and further validation in clinical studies.

**Figure 2:**
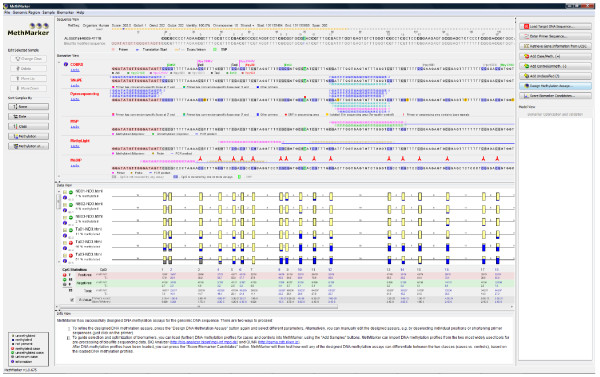
**This figure shows a screenshot of MethMarker's main analysis window**. From top to bottom, MethMarker displays gene annotation data for the region of interest; its genomic DNA sequence as well as the bisulfite converted sequence; automatically generated assays for the supported experimental methods (COBRA, bisulfite SNuPE, bisulfite pyrosequencing, MSP, MethyLight and MeDIP-qPCR); DNA methylation information for the region of interest, which has been loaded into MethMarker (yellow bars correspond to unmethylated CpGs, blue bars to methylated CpGs); a statistical summary of CpG positions within the region of interest; and - at the bottom - a text field providing advice for the user. All views are highly interactive and can be adjusted to control MethMarker's behavior.

Apart from two key experimental analyses - the generation of high-resolution DNA methylation data in step one and assay validation in step six - this workflow is essentially bioinformatic in nature. We developed the MethMarker software as a user-friendly implementation of the bioinformatic steps, including automatic assay design for six widely used experimental methods (COBRA, bisulfite SNuPE, bisulfite pyrosequencing, MethyLight, MSP and MeDIP-qPCR) and computational biomarker optimization. MethMarker integrates well with existing bioinformatic tools for analyzing DNA methylation (reviewed in [17]): epigenome analysis tools such as Galaxy [18] and EpiGRAPH [19] can be used to select promising DMRs for optimization with MethMarker, and high-resolution DNA methylation data can be imported directly from three widely used software packages, BiQ Analyzer [20], QUMA [21] and EpiTYPER [22], as well as from custom tables. Finally, optimized biomarkers can be exported in the standardized predictive model markup language (PMML) format [23], which facilitates interoperation with molecular diagnostics software. A typical screenshot of MethMarker is displayed in Figure 2.

## Application

To illustrate the biomarker development workflow outlined in Figure 1 and to demonstrate the practical use of MethMarker, we describe its application to the *MGMT *gene promoter, highlighting important decisions, necessary validation experiments and potential stumbling blocks. The raw data for this case study are taken from a recent experimental study [15] and are included as a demonstration dataset in the MethMarker download package.

The *MGMT *gene encodes a DNA repair protein, which removes alkyl groups from the *O*^6^-position of guanine, therefore protecting the DNA from accumulating excessive damage [24]. It has been shown in a number of studies (see [25] and references therein) that hypermethylation of the *MGMT *promoter is a frequent event in various cancers (that is, is relevant for diagnosis), that it is associated with decreased survival if the cancer is untreated (that is, is relevant for prognosis), and that it renders tumors susceptible to alkylating drugs such as temozolomide (that is, is also relevant for therapy optimization). However, until recently no assay for measuring *MGMT *promoter methylation had been available that was robust enough for routine clinical use and fully compatible with DNA extracted from formalin-fixed, paraffin-embedded samples [26].

For these reasons, the promoter of the *MGMT *gene is an excellent target region for demonstrating the systematic development of a DNA methylation biomarker, such that the resulting assay is accurate, robust and cost-efficient enough for clinical use. To start with, we obtain the genomic DNA sequence of the *MGMT *promoter region from the UCSC Genome Browser [27]. We also obtain 22 glioblastoma samples, a subset of them showing *MGMT *promoter methylation, as well as three normal brain samples for use as healthy tissue controls. Next, bisulfite-specific PCR primers are designed (manually or using a software tool such as Methyl Primer Express [28]), and clonal bisulfite sequencing is performed on DNA from all samples according to a widely used protocol [29]. The sequencing data are processed and quality controlled with BiQ Analyzer [20], resulting in 25 high-resolution DNA methylation profiles that are used as training samples. (Note that it is usually sufficient to have five to ten training samples per class to guide the optimization step. In our case, however, it was not clear *a priori *how many of the tumor samples would turn out to belong to the methylated cases or to the unmethylated controls, respectively. Hence, a relatively large number of samples were subjected to clonal bisulfite sequencing.)

Next, the genome sequence of the target region, the corresponding primer sequences and the BiQ-Analyzer processed DNA methylation profiles are imported into MethMarker. The software tool automatically identifies the correct location of the *MGMT *promoter on human chromosome 10, visualizes the position of the first exon and aligns the DNA methylation profiles of all 25 training samples (Figure 2). We let MethMarker classify the training samples into cases and controls, using hierarchical clustering of the DNA methylation profiles. Consistent with previous observations, we obtain a large cluster of samples in which the *MGMT *promoter is unmethylated and a smaller cluster consisting of tumor samples with methylated *MGMT *promoters. The former cluster - which we will refer to as 'controls' - contains the normal brain samples and a subset of tumors that are likely to be resistant to alkylating agents used for chemotherapy. The latter cluster ('cases') comprises tumor samples only, presumably those that are susceptible for chemotherapy using alkylating drugs such as temozolomide [30].

Based on this classification, our goal is to find a DNA methylation assay (or a combination of several assays) that provides accurate, robust and cost-efficient separation between cases and controls. First, we let MethMarker design all feasible DNA methylation assays for the target region, using COBRA, bisulfite SNuPE, bisulfite pyrosequencing, MethyLight and MeDIP-qPCR. We chose to exclude MSP because several MSP-based assays for *MGMT *promoter hypermethylation are already available [26] and because MSP-based assays do not always work well on formalin-fixed, paraffin-embedded samples [15]. Next, we let MethMarker score the individual assays in terms of their correlation with the overall DNA methylation level in each of the training samples (Additional data file 1). A Pearson correlation coefficient above 0.9 and a Spearman correlation coefficient above 0.8 indicate a highly accurate and predictive assay. Even when a single CpG site already provides a highly accurate measurement - as is the case here - it is highly recommended to use a combination of at least three to four CpG sites in order to increase robustness of the DNA methylation assay in the presence of experimental noise and rare sequence polymorphisms. To that end, MethMarker identifies the optimal combinations of DNA methylation assays for each method, again ranked by their correlation with the overall DNA methylation level in each of the training samples (Additional data file 1).

**Figure 3:**
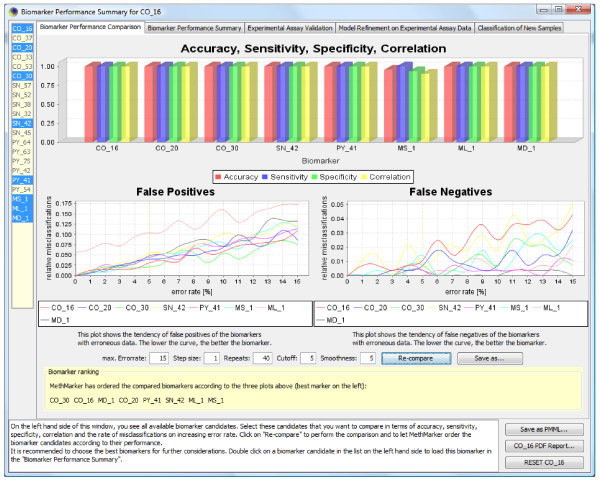
**This figure displays a screenshot of MethMarker's biomarker performance comparison, assessing the robustness of candidate biomarkers to elevated error rates**. In this example, CO_30 and CO_16 exhibit the overall best performance, in terms of low false positive/negative rates as well as high levels of accuracy, sensitivity, specificity and correlation.

From the resulting list, we select several assay combinations that appear to provide a suitable balance between accuracy, robustness and cost (higher robustness is usually achieved by including more CpG sites, which makes the candidate biomarker more expensive to use). For each of these assay combinations, we let MethMarker optimize logistic regression models that predict whether a sample belongs to the cases or to the controls (Figure 3). During this step, weights are learned for the individual assays in order to maximize the classification accuracy of the candidate biomarker. MethMarker benchmarks the candidate biomarkers in terms of accuracy, correlation, specificity and sensitivity. Additionally, the biomarkers' robustness is assessed by comparing false positive and false negative rates under increasing error rate, by simulating noisy measurement data. This step accounts for the fact that not all error sources may be well-represented in the training data. For example, COBRA, bisulfite SNuPE and bisulfite pyrosequencing are sensitive to rare inherited C-to-T single nucleotide polymorphisms at the assayed CpGs, and MSP as well as MethyLight can give rise to erroneous measurements if the DNA methylation profile in the target region only partially matches with the designed probe (see Mikeska *et al*. [15] and Bock *et al*. [20] for a more in-depth discussion of potential error sources).

**Figure 4:**
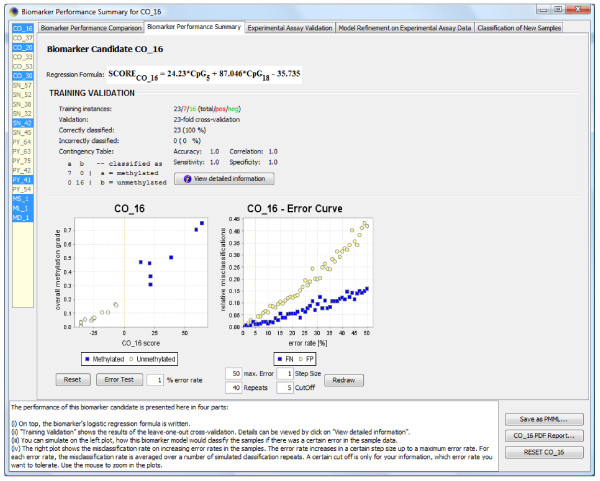
**A screenshot of MethMarker's performance window, summarizing the evaluation of a bisulfite pyrosequencing-based biomarker**. In the upper panel, MethMarker displays the optimized regression formula, which predicts - based on measurement values for CpGs number 5 and 18 - whether a sample belongs to the case (that is, is methylated, indicated by positive score values) or to the control group (that is, is unmethylated, indicated by negative score values). Note that the score value is a measure of the probability with which the sample is a case rather than a control, not an estimate of the DNA methylation level (in fact, the probability *p *can be calculated from the score *s *with a simple formula: . The center panel displays the results of leave-one-out cross-validation, providing an estimate of the biomarker performance on new data. The diagrams at the bottom visualize the degree of separation between the two classes when plotting the measured level of DNA methylation over the score value of the regression formula (left) and the robustness of predictions in the face of increasing noise levels (right).

For each candidate biomarker, MethMarker also calculates an extensive performance evaluation summary (Figure 4). We use the results from this window to compare how well several top-scoring candidate biomarkers separate between the methylated cases and unmethylated controls. Also, we test the robustness of each candidate biomarker by artificially introducing noise and observing how much noise it can tolerate until the first classification errors start to appear. As a quintessence of all performance evaluations of MethMarker, we conclude that the following two candidate biomarkers are most suitable for assessing promoter hypermethylation of the *MGMT *gene in routine clinical use: the COBRA biomarker comprising CpG sites 5/6 and 18, utilizing the *Hpy99I *and *HpyCH4III *restriction endonucleases (*r *= 0.985), and the bisulfite pyrosequencing biomarker comprising CpG sites 13, 18 and 20 (*r *= 0.990). Both biomarkers achieve 100% test set accuracy during leave-one-out cross-validation. Compared to the biomarkers that we previously established for the same dataset [15], the biomarkers identified by MethMarker achieve an identical accuracy and score marginally higher in terms of correlation and robustness (data not shown). Nevertheless, we recommend that practical studies of *MGMT *promoter methylation continue using the previously published biomarkers [15] because they have been validated experimentally, while the two MethMarker-derived biomarkers reported here have not been tested on clinical samples.

Having completed the design, optimization and computational validation of candidate biomarker DNA methylation assay for the *MGMT *promoter, two key steps remain: experimental assay validation and experimental biomarker validation. First, it is essential to make sure that the DNA methylation assays included in the selected biomarker work well in the lab and result in roughly the same DNA methylation measurements as predicted based on the high-resolution DNA methylation profiles. To that end, the assays are applied to DNA from the training samples, and each assay's empirical measurement value is compared with the simulated measurement value that MethMarker calculated from the high-resolution profiles. Assays showing low correlation or high deviation should be rejected from practical use as biomarkers. Second, the most important step for any new DNA methylation biomarker is to validate its sensitivity, specificity and practical utility in a large number of patients, both by retrospective studies based on archival material with known clinical history and in prospective clinical trials. While several clinical trials have already confirmed the effect of *MGMT *hypermethylation on chemotherapy resistance in gliomas [31] and glioblastomas [30,32], the MethMarker-optimized biomarker may facilitate the clinical confirmation of *MGMT*'s predictive role in other cancers.

## Conclusions

Recent advances in genome-wide DNA methylation mapping have provided researchers with rapid and cost-efficient ways to contribute to the ever-growing list of genomic regions reported as differentially methylated in specific cancers and/or patient subgroups. However, a comparable advance for the efficient conversion of DMRs into clinical biomarkers is lacking. Thus, the rate with which new DNA methylation biomarkers are tested and confirmed in clinical trials has remained disappointingly low. While it is inevitable that a large percentage of candidate biomarkers will fail in clinical trials (either because they are not reproducible in different patient cohorts or because their sensitivity and specificity are insufficient for practical use), a more systematic approach to epigenetic biomarker development could help discard many of these unsuccessful candidates early and at low cost. Conversely, careful selection and optimization of candidate biomarkers can reduce the risk of losing effective biomarkers due to contingencies of the validation process, such as accidental selection of DNA methylation assays that measure highly noisy CpG positions in a promoter region that would otherwise provide reliable classification. The workflow described in this paper provides a starting point toward a more systematic way of transforming disease-specific DMRs into robust and cost-efficient clinical biomarkers. The MethMarker software was developed to facilitate the implementation of this workflow. To enable further refinement and adaptation to local requirements, we are happy to share MethMarker's source code with interested researchers.

## Materials and methods

MethMarker is implemented in Java (version 1.5 or later required). It is platform-independent and can be launched directly from within a web browser. The software comes with a case-study tutorial demonstrating the design, optimization and validation of a DNA methylation biomarker based on the *MGMT *gene. MethMarker's user interface reflects the workflow for biomarker design, optimization and validation outlined in Figure 1.

### Step 1: data import

As the first step, the DMR of interest is imported. MethMarker supports several sequence formats, including FASTA, GenBank and EMBL. Typical regions of interest include the promoters of tumor suppressor genes and CpG islands that exhibit cancer-specific hypermethylation. However, MethMarker imposes no restrictions on the type of region to be analyzed. MethMarker can thus be applied not only to human cancers, but more generally to epigenotyping in all kinds of organisms that exhibit CpG dinucleotide methylation.

High-resolution DNA methylation profiles for a subset of cases and controls are crucial for MethMarker's optimization process, as they provide the training set on which all candidate biomarkers are optimized and computationally validated. These profiles are usually derived by clonal bisulfite sequencing [33] or mass spectrometry and preprocessed with appropriate tools. MethMarker can directly import DNA methylation profiles from files generated with BiQ Analyzer [20], QUMA [21] and EpiTYPER [22], and it is easy to convert DNA methylation data from a different source into a format that can be read by MethMarker.

On completion of data import, MethMarker displays a high-resolution DNA methylation profile of the region of interest, visualized as lollipop diagrams or as methylation propensity diagrams. Internally, MethMarker uses Needleman-Wunsch sequence alignment [34] in order to correct for incomplete overlap between the target region and the DNA methylation profiles. It is thus possible to tile a large target region with several bisulfite sequencing amplicons.

Optionally, MethMarker can annotate the region with transcription start site and exon positions retrieved from the UCSC Genome Browser [27]. To that end, MethMarker performs an automatic BLAT search on the UCSC Genome Browser website, obtains the genomic coordinates of the region and retrieves exon information for overlapping RefGene genes from the UCSC Table Browser. Data on single nucleotide polymorphisms are acquired in the same way, enabling MethMarker to avoid polymorphic sites when designing DNA methylation assays. All annotation data can be manually revised and amended.

### Step 2: design of DNA methylation assays

MethMarker implements automatic assay design for six experimental methods commonly used for DNA methylation analysis: COBRA, bisulfite SNuPE, bisulfite pyrosequencing, MethyLight, MSP and MeDIP-qPCR. The first five methods utilize bisulfite treatment of genomic DNA to detect DNA methylation indirectly. However, they differ in the way they interrogate the amount of DNA methylation, leading to specific experimental constraints that limit the application of each method to assaying a subset of CpG positions. The sixth method, MeDIP-qPCR, uses an antibody-based approach to enrich for methylated genomic DNA, which leads to quite different experimental constraints [35]. For all methods, assay design rules were developed, reviewed by domain experts and implemented in MethMarker, as described in more detail in the MethMarker assay design dialogue. However, it is recommended that all primers designed with MethMarker are reviewed by the experimenter before ordering, to exclude problems such as hairpins, self-dimers and cross-dimers, which MethMarker does not automatically check for.

All automatically designed DNA methylation assays can be visualized, revised or excluded by the user, for example, based on results of previous experiments. Furthermore, MethMarker allows users to define and incorporate custom assays, which enables the software to include experimental methods that are not directly supported.

### Step 3: scoring of DNA methylation assays

Based on the samples for which high-resolution DNA methylation profiles are available (see step 1), MethMarker scores all DNA methylation assays in terms of their correlation with the overall level of DNA methylation in each sample. The measurement values of the DNA methylation assays are calculated directly from the high-resolution DNA methylation profiles, using a set of method-specific rules. For COBRA, bisulfite SNuPE and bisulfite pyrosequencing, the measurement value is calculated simply as the average DNA methylation level of the assayed CpG site(s), based on the high-resolution DNA methylation profiles of the respective sample. For MSP, MethyLight and MeDIP-qPCR, the measurement value is calculated as the percentage of individual clones in which all participating CpG sites are simultaneously methylated. To better resemble real PCR conditions, for MSP and MethyLight a single CpG position is allowed to have an incorrect methylation value. While simulated measurements cannot replace experimental validation of the resulting DNA methylation assays (see [36] for a discussion of the limitations of simulating DNA methylation measurements *in silico*), they provide a suitable indication for identifying the most predictive DNA methylation assays to be included in the optimization step.

### Step 4: biomarker optimization

From the list of DNA methylation assays, ranked by their correlation with the overall DNA methylation levels of the training samples, the user can select a subset for biomarker optimization. MethMarker then scores all possible combinations of the selected DNA methylation assays and again assesses the correlation with the overall DNA methylation levels of the training samples. To allow for fair comparison between assay sets of different sizes, no weight fitting is performed at this stage. Rather, the score value of each combination is calculated as the mean measurement value of all contributing DNA methylation assays. The results of this comparison are listed in the order of decreasing correlation coefficients, and the user can select a subset of the most highly scoring combinations of DNA methylation assays for optimization and computational validation as candidate biomarkers, a procedure that is performed as follows.

First, the training samples are classified into cases and controls. This classification can be performed based on known sample information (for example, tumor samples versus normal tissue annotation) or based on the DNA methylation profiles themselves, using one of the following methods: a fixed threshold on the average DNA methylation level, hierarchical clustering, or *K*-means clustering with *K *= 2. In all cases, the DNA methylation profiles in the subset with the higher average methylation levels are labeled as methylated 'cases' and the remaining profiles are labeled as unmethylated 'controls'.

Second, logistic regression is used to optimize the weight with which the individual measurements contribute to the overall biomarker score, accounting for the fact that different CpGs vary in their predictiveness of the overall level of DNA methylation. Internally, MethMarker uses the WEKA package [37] to train a logistic regression model for each candidate biomarker, classifying the training samples into cases versus controls based on simulated methylation measurements for all contributing CpGs.

Third, the predictiveness of the logistic regression models is validated by leave-one-out cross-validation - that is, the logistic regression models are repeatedly trained on all but one training samples and their prediction performance is assessed on the remaining sample. The results of the optimization step, including a cross-validation-based estimate of the prediction performance on new data, are displayed in the biomarker summary window (Figure 4).

### Step 5: validation of DNA methylation biomarkers

While the results of the leave-one-out cross-validation (step 4) already provide an important selection criterion for identifying the most suitable DNA methylation biomarkers, they do not account for potential errors and experimental problems that can occur during practical use. MethMarker therefore provides an additional validation step, which assesses the robustness of each candidate biomarker toward noisy data, sequencing errors and unknown single nucleotide polymorphisms. In this step, the optimal logistic regression model is re-applied to all samples for which high-resolution DNA methylation profiles are available (this can include samples that were not taken into account in the training phase - for example, because they constitute outliers or borderline cases), and the biomarker's prediction confidence for a given sample is plotted against its mean DNA methylation level, as calculated from the DNA methylation profiles. It is thus possible to visually assess how well each candidate biomarker separates between the (methylated) cases and (unmethylated) controls. Furthermore, MethMarker assesses the robustness toward erroneous data - such as sequencing errors or unknown single nucleotide polymorphisms - by randomly changing the DNA methylation measurement of a subset of CpGs. The error rate is varied over a wide range, and the impact on the prediction accuracy is visualized in the biomarker summary window (Figure 4), enabling the user to assess whether or not a specific candidate biomarker is sufficiently robust for clinical use.

### Step 6: application of DNA methylation biomarkers

Based on the results of the computational assessment, the user selects a few of the most promising biomarkers for experimental validation, performs the necessary DNA methylation assays on DNA from the training samples and uploads the results into MethMarker. By comparison between the simulated and actual measurements, MethMarker can evaluate the reliability of each candidate biomarker under routine experimental conditions and re-train its logistic regression models accordingly (for example, down-weighting the contribution of a CpG whose DNA methylation assay exhibits a high level of experimental noise). This experimental validation step is important because it corrects for any deviations from the theoretically optimal measurement conditions that underlie the computational simulation of measurement values.

When the optimization and validation steps are completed and the user is satisfied with the overall performance, one or more candidate biomarkers are typically selected for further development. MethMarker provides two ways of facilitating the steps toward comprehensive clinical testing and widespread practical use. First, MethMarker can generate a comprehensive PDF report describing the key properties of a selected biomarker. This report includes the final sample classification formula as well as a summary of the accuracy and robustness assessment. Based on this file, it is straightforward to apply the biomarker assay to new data, requiring no statistical or bioinformatic tools beyond a pocket calculator. Second, a selected biomarker can be exported in a standardized data format, PMML, which is supported by several statistics packages and can be imported into diagnostics software. PMML has been developed by the Data Mining Group [23] to facilitate data exchange between developers and users of classification and regression models. All classifiers created with MethMarker fulfill the PMML 3.2 standard (see Additional data file 2 for illustration). Third, MethMarker supports multi-center biomarker validation studies. To that end, the PDF and PMML documentation files of the selected biomarker are distributed to all participating centers; each center then performs the necessary DNA methylation assays for all local samples, loads the PMML file and the measurement values into MethMarker and obtains the biomarker result for each of their samples; finally, the measurement values from all centers as well as the corresponding clinical data are combined, loaded into MethMarker and a global assessment of biomarker performance is obtained. If the performance is not satisfactory, the entire process can be reiterated and the biomarker re-optimized based on the data obtained in the previous round of validations.

## Abbreviations

COBRA: combined bisulfite restriction analysis; DMR: differentially methylated region; MeDIP-qPCR: methylated DNA immunoprecipitation quantitative PCR; MGMT: *O*^6^-methylguanine DNA methyltransferase; MSP: methylation-specific polymerase chain reaction; PMML: predictive model markup language; SNuPE: single-nucleotide primer extension.

## Competing interests

The authors declare that they have no competing interests.

## Authors' contributions

CB initiated the project and conceptualized workflow and software. PS designed and implemented MethMarker, developed the case study tutorial, set up the website and drafted the paper. TM devised the assay design rules and contributed his experience with COBRA, bisulfite SNuPE, bisulfite Pyrosequencing, MSP, MethyLight and MeDIP-qPCR. He also provided experimental data and performed extensive beta testing. AW provided experimental data. TL contributed advice and ideas throughout the project. All authors were involved in the writing of the paper.

## Additional data files

The following additional data are available with the online version of this paper: a screenshot of MethMarker's performance ranking of DNA methylation assays and candidate biomarkers (Additional data file 1); the XML-based PMML model that MethMarker uses for exporting, importing and storing candidate biomarkers (Additional data file 2).

## Supplementary Material

Additional data file 1 Screenshot of MethMarker's performance ranking of DNA methylation assays and candidate biomarkers. Click here for file

Additional data file 2 The XML-based PMML model that MethMarker uses for exporting, importing and storing candidate biomarkers. Click here for file
