# Supplementary material for: MethMarker: user-friendly design and optimization of gene-specific DNA methylation assays
Source: Genome Biol. 2009 Oct 5;10(10):R105. doi: 10.1186/gb-2009-10-10-r105 (PMC2784320; doi:10.1186/gb-2009-10-10-r105)
Supplement: Additional data file 1 — Screenshot of MethMarker's performance ranking of DNA methylation assays and candidate biomarkers. [file gb-2009-10-10-r105-S1.PDF]

Additional data file 1:

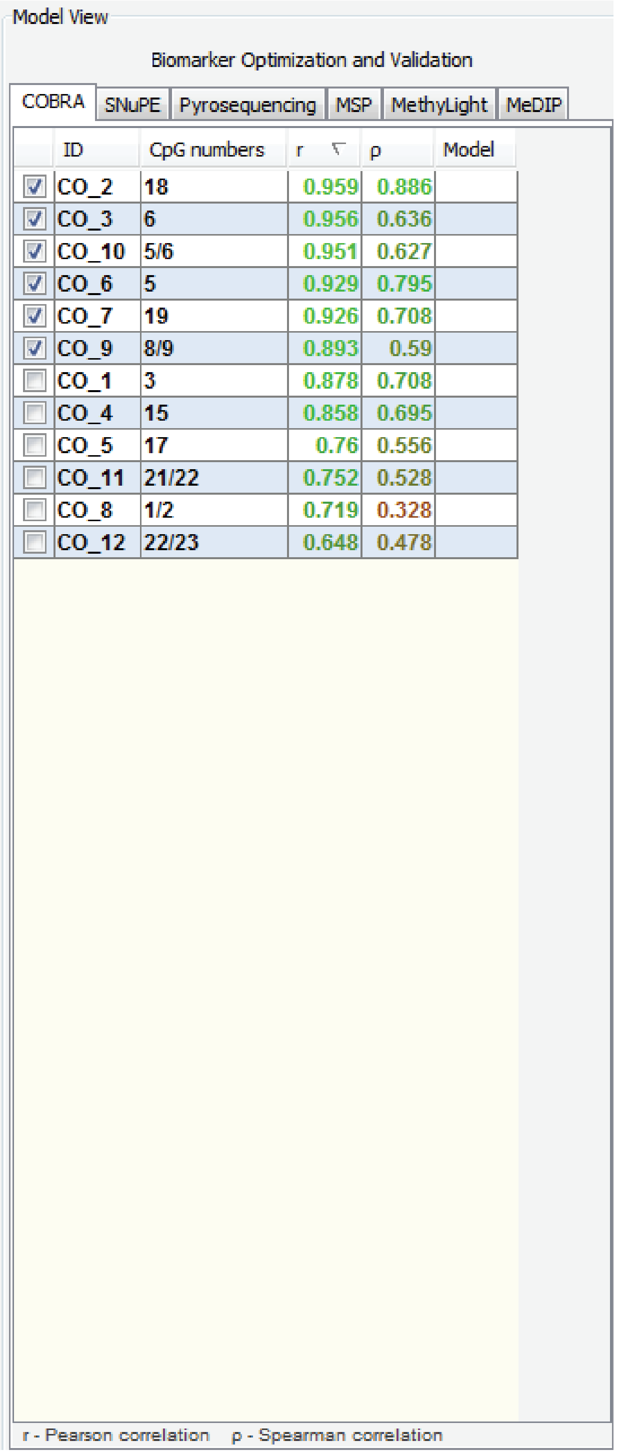

Model View

Biomarker Optimization and Validation

COBRA | SNuPE | Pyrosequencing | MSP | MethyLight | MeDIP

	ID	CpG numbers	r	ρ	Model
☑	CO_2	18	0.959	0.886	
☑	CO_3	6	0.956	0.636	
☑	CO_10	5/6	0.951	0.627	
☑	CO_6	5	0.929	0.795	
☑	CO_7	19	0.926	0.708	
☑	CO_9	8/9	0.893	0.59	
☐	CO_1	3	0.878	0.708	
☐	CO_4	15	0.858	0.695	
☐	CO_5	17	0.76	0.556	
☐	CO_11	21/22	0.752	0.528	
☐	CO_8	1/2	0.719	0.328	
☐	CO_12	22/23	0.648	0.478	

r - Pearson correlation   ρ - Spearman correlation

A)

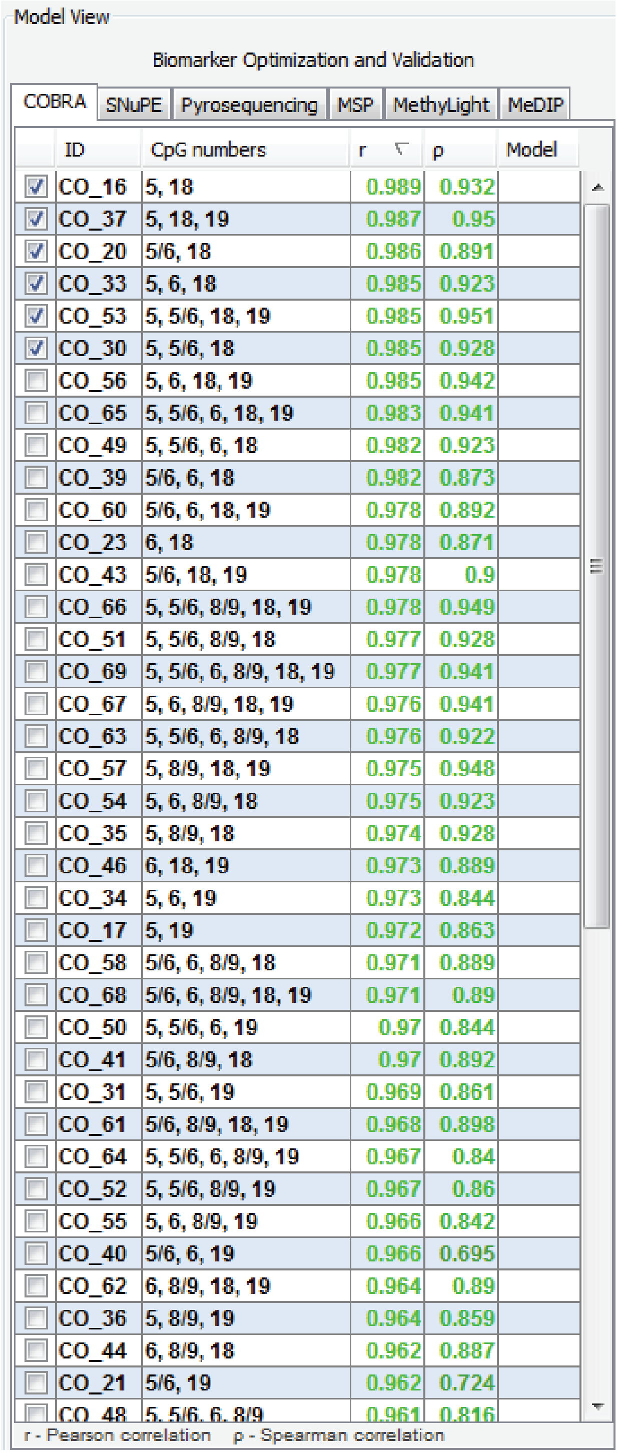

Model View

Biomarker Optimization and Validation

COBRA | SNuPE | Pyrosequencing | MSP | MethyLight | MeDIP

	ID	CpG numbers	r	ρ	Model
☑	CO_16	5, 18	0.989	0.932	
☑	CO_37	5, 18, 19	0.987	0.95	
☑	CO_20	5/6, 18	0.986	0.891	
☑	CO_33	5, 6, 18	0.985	0.923	
☑	CO_53	5, 5/6, 18, 19	0.985	0.951	
☑	CO_30	5, 5/6, 18	0.985	0.928	
☐	CO_56	5, 6, 18, 19	0.985	0.942	
☐	CO_65	5, 5/6, 6, 18, 19	0.983	0.941	
☐	CO_49	5, 5/6, 6, 18	0.982	0.923	
☐	CO_39	5/6, 6, 18	0.982	0.873	
☐	CO_60	5/6, 6, 18, 19	0.978	0.892	
☐	CO_23	6, 18	0.978	0.871	
☐	CO_43	5/6, 18, 19	0.978	0.9	
☐	CO_66	5, 5/6, 8/9, 18, 19	0.978	0.949	
☐	CO_51	5, 5/6, 8/9, 18	0.977	0.928	
☐	CO_69	5, 5/6, 6, 8/9, 18, 19	0.977	0.941	
☐	CO_67	5, 6, 8/9, 18, 19	0.976	0.941	
☐	CO_63	5, 5/6, 6, 8/9, 18	0.976	0.922	
☐	CO_57	5, 8/9, 18, 19	0.975	0.948	
☐	CO_54	5, 6, 8/9, 18	0.975	0.923	
☐	CO_35	5, 8/9, 18	0.974	0.928	
☐	CO_46	6, 18, 19	0.973	0.889	
☐	CO_34	5, 6, 19	0.973	0.844	
☐	CO_17	5, 19	0.972	0.863	
☐	CO_58	5/6, 6, 8/9, 18	0.971	0.889	
☐	CO_68	5/6, 6, 8/9, 18, 19	0.971	0.89	
☐	CO_50	5, 5/6, 6, 19	0.97	0.844	
☐	CO_41	5/6, 8/9, 18	0.97	0.892	
☐	CO_31	5, 5/6, 19	0.969	0.861	
☐	CO_61	5/6, 8/9, 18, 19	0.968	0.898	
☐	CO_64	5, 5/6, 6, 8/9, 19	0.967	0.84	
☐	CO_52	5, 5/6, 8/9, 19	0.967	0.86	
☐	CO_55	5, 6, 8/9, 19	0.966	0.842	
☐	CO_40	5/6, 6, 19	0.966	0.695	
☐	CO_62	6, 8/9, 18, 19	0.964	0.89	
☐	CO_36	5, 8/9, 19	0.964	0.859	
☐	CO_44	6, 8/9, 18	0.962	0.887	
☐	CO_21	5/6, 19	0.962	0.724	
☐	CO_48	5, 5/6, 6, 8/9	0.961	0.816	

r - Pearson correlation   ρ - Spearman correlation

B)

This figure displays a screenshot of MethMarker's performance ranking of DNA methylation assays (A) and candidate biomarkers (B). While each DNA methylation assay corresponds to a single measurement, candidate biomarkers combine several DNA methylation assays in order to achieve increased robustness and accuracy. Performance is scored by the degree of correlation – as measured by Pearson's r and Spearman's ρ correlation coefficients – between the score value and the overall DNA methylation level across all samples. For DNA methylation assays, the score value is identical to the DNA methylation read-out. For candidate biomarkers combining the several DNA methylation assays, the score value is calculated as the mean DNA methylation read-out of all contributing assays. No weight fitting is performed at this stage in order to allow for fair comparison between candidate biomarkers of different complexity.
