# Supplementary material for: MethMarker: user-friendly design and optimization of gene-specific DNA methylation assays
Source: Genome Biol. 2009 Oct 5;10(10):R105. doi: 10.1186/gb-2009-10-10-r105 (PMC2784320; doi:10.1186/gb-2009-10-10-r105)
Supplement: Additional data file 2 — The XML-based PMML model that MethMarker uses for exporting, importing and storing candidate biomarkers. [file gb-2009-10-10-r105-S2.PDF]

Additional data file 2:


```
- <PMML version="3.2">
  - <Header copyright="DMG.org">
      <Application name="MethMarker"/>
    - <Annotation>
        <Extension description="name">55531|nt46891-47156</Extension>
      - <Extension description="sequence">
          CGCGCCCCTAGAACGCTTTGCGTCCCGACGCCCGCAGGTCCTCGCGGTGCGRACCGTTTGCGACTTGGTGAGTGTCTGGGTCGCCTCGCTCCCGGAAGA
        </Extension>
        <Extension description="primer3">TTTAGGTGTTGTTTAGTTT</Extension>
        <Extension description="primer5">GGATATGTTGGGATAGTT</Extension>
      </Annotation>
    </Header>
  - <DataDictionary numberOfFields="4">
      <DataField name="CpG-11-" optype="continuous" dataType="double"/>
      <DataField name="CpG-13-" optype="continuous" dataType="double"/>
      <DataField name="CpG-20-" optype="continuous" dataType="double"/>
    - <DataField name="class" optype="categorical" dataType="string">
        <Value value="methylated"/>
        <Value value="unmethylated"/>
      </DataField>
    </DataDictionary>
  - <RegressionModel modelName="PY_42" algorithmName="Logistic Regression" functionName="regression" normalizationMethod="softmax" targetFieldName="class">
    - <MiningSchema>
        <MiningField name="CpG-11-"/>
        <MiningField name="CpG-13-"/>
        <MiningField name="CpG-20-"/>
        <MiningField name="class" usageType="predicted"/>
      </MiningSchema>
    - <RegressionTable targetCategory="no" intercept="-31.224215470714434">
        <NumericPredictor name="CpG-11-" coefficient="31.985407642372028"/>
        <NumericPredictor name="CpG-13-" coefficient="46.68942168442977"/>
        <NumericPredictor name="CpG-20-" coefficient="34.00776139565338"/>
      </RegressionTable>
    </RegressionModel>
  </PMML>
```


This figure illustrates the XML-based predictive model markup language (PMML) model that MethMarker uses for exporting, importing and storing candidate biomarkers. The model file includes the DNA sequence of the corresponding genomic region, primer information, a list of assayed CpG sites and the coefficients of the optimized prediction model. The file meets the specifications of the Data Mining Group's PMML format [http://www.dmg.org/pmml-v3-2.html].
